# Review of Methods for Evaluating Changes in the Tension and Properties of the Gluteus Medius Muscle (GMED) and the Tensor Fascia Latae (TFL) as a Result of Hip Osteoarthritis (HOA) and After Total Hip Arthroplasty (THA)—Could MyotonPRO Assessment Be the New Standard?

**DOI:** 10.3390/jcm14175982

**Published:** 2025-08-24

**Authors:** Agnieszka Posturzyńska, Artur Łazorko, Bartosz Cukierman, Agnieszka Tomczyk-Warunek, Anna Winiarska, Tomasz Skrzypek, Magdalena Lis, Jaromir Jarecki

**Affiliations:** 1 Department of Traumatology, Orthopedics and Rehabilitation, Medical University of Lublin, 20-954 Lublin, Poland; agnieszka.posturzynska@umlub.pl; 2 Department of Orthopedics and Traumatology, Holy Family Hospital, 36-060 Głogów Małopolski, Poland; lazorko@wp.pl; 3 Department of Trauma and Orthopedic Surgery, Independent Public Health Care Facility in Sokółka, St. Gen. Wł. Sikorskiego 40, 16-100 Sokółka, Poland; bartoszcukierman@gmail.com; 4 Institute of Animal Nutrition and Bromatology, Department of Bromatology and Nutrition Physiology, University of Life Sciences in Lublin, Akademicka 13, 20-950 Lublin, Poland; anna.mieczan@up.lublin.pl; 5 Department of Biomedicine and Environmental Research, Faculty of Medicine, John Paul II Catholic University of Lublin, 20-708 Lublin, Poland; tomasz.skrzypek@kul.pl (T.S.); magdalena.lis@kul.pl (M.L.)

**Keywords:** MyotonPRO, gluteus medius muscle, tensor fasciae late, total hip arthroplasty, osteoarthritis, hip osteoarthritis

## Abstract

**Background/Objectives:** Osteoarthritis (OA) is a condition affecting many joints, including the hip. The treatment of advanced hip osteoarthritis (HOA) involves total hip arthroplasty (THA). Atrophy of abductor muscles is often diagnosed in patients with HOA. This review presents a number of studies evaluating changes that occur in the gluteus medius (GMED) and tensor fasciae latae (TFL) as a result of HOA and THA. MyotonPRO is a portable and non-invasive device that allows for the assessment of muscle quality. This review aimed to collect studies assessing changes in GMED and TFL following HOA and THA and to determine whether MyotonPRO can be used for this assessment. **Methods:** We conducted a comprehensive search of databases, including Google Scholar, Science Direct, and PubMed, for relevant articles published between 2012 and 2024. A total of 37 articles were included in our review. Qualified papers evaluated changes in the lower limb muscles, including TFL and GMED, as a result of HOA and THA using MyotonPRO and other methods. **Results:** In this article, we emphasize the influence of the tested muscles on HOA and the postoperative course after THA using MyotonPRO. We have shown that myotonPRO was used to assess muscle changes due to knee OA and GMED and TFL in other groups of patients. **Conclusions:** This is the first review of the literature to indicate a new direction of research using myotonPRO. The use of MyotonPRO will allow for the more detailed development of rehabilitation programs for patients with HOA and after THA.

## 1. Introduction

Osteoarthritis (OA) is a condition affecting many joints, including the knee and hip. It is one of the most common causes of disability worldwide (11th place) [1,2]. Hip osteoarthritis (HOA) is the second most common form of osteoarthritis after knee osteoarthritis (KOA) [3,4]. As in the case of other forms of OA, the incidence of HOA is expected to increase, given the phenomenon of population aging and the growing epidemic of obesity [5]. This disease not only induces the development of negative changes in cartilage and bone tissue but also causes muscle weakness and proprioceptive disorders with balance impairment [2]. According to epidemiological data, in 2017, the prevalence of HOA was estimated to be 40 million people worldwide, and the incidence of this disease was estimated to be two million patients [6].

Weakness of muscles surrounding an HOA-affected joint is associated with their atrophy. Studies have also suggested that muscle weakness may promote the development, progression, and severity of HOA in the surrounding joints [5].

The abductor is one of the muscles surrounding the hip joint. It is responsible not only for movement but also for stabilization of this joint [5,7]. Atrophy of abductor muscles is often diagnosed in patients with HOA. Knowledge of the function of these muscles is essential for accurate and correct diagnosis, facilitating the application of appropriate patient-tailored HOA treatment [8]. This group of muscles includes gluteus medius (GMED), gluteus minimus (GMIN), and tensor fasciae latae (TFL). Other muscles, such as the upper part of the gluteus maximus (GMAX), sartorius, and piriformis, are also included in the group of abductors, but they serve secondary functions. GMED and TFL are among the largest abductor muscles [9].

In recent years, the number of studies assessing changes in the activity, function, and size of the hip abductor muscles in HOA has increased. These investigations suggest that the activity of this muscle group changes considerably during HOA. However, some studies have reported a decrease in the activity of these muscles, while other investigations have shown their enhanced activity [10,11,12,13] or no changes at all [7,11]. Nevertheless, the biomechanical properties (stiffness and elasticity), viscoelasticity, and tension of abductor muscles in HOA are still being evaluated [14,15,16,17] using the MyotonPRO device (Myoton AS, Talinn, Estopnia).

Since the etiology of hip osteoarthritis is not fully understood, treatment of the disease is limited. At present, only symptomatic treatment is available. The treatment of advanced hip osteoarthritis involves surgical intervention based on total hip arthroplasty (THA), which is performed using various surgical approaches [18,19].

Hence, researchers are currently investigating THA-induced changes in the activity and strength of abductor muscles. Only a few studies have shown that the weakness and atrophy of abductor muscles observed in the development of HOA continue after THA. At 6 months post-THA, the strength of this muscle group improved by only 50%, which affected the recovery process [20,21]. The biomechanics of the hip joint post THA depend on the proper function of the muscles, which are responsible for the proper long-term functioning of the endoprosthesis [22]. As in the case of HOA, no studies have assessed changes in muscle stiffness, elasticity, and tension after THA. Similarly, no studies have analyzed the use of the MyotonPRO device [14,15,16,17].

MyotonPRO is a portable, non-invasive myotometry device. Compared to other methods used to assess muscle quality (elastography or EMG—electromyography), the device is relatively easy to use, and the examination is relatively quick [14]. It can be performed under various conditions and does not require high qualifications, as only basic training is sufficient [23]. Additionally, this examination is cheaper than elastography or EMG because no special laboratory conditions are required. Recent reports have indicated that MyotonPRO examination results are credible and reliable. Their reliability has been demonstrated in examinations of the gastrocnemius muscle [24], quadriceps [14,15,25,26], and tendons in tendinopathies [27,28]. Several clinical studies have also confirmed the suitability of MyotonPRO for assessing muscle and tendon quality in various disease entities, such as paratonia in dementia [29], Parkinson’s disease [30], and stroke [31]. The clinical value of MyotonPRO and its usefulness for monitoring treatment in stroke patients have also been confirmed [32,33].

**Figure 1:**
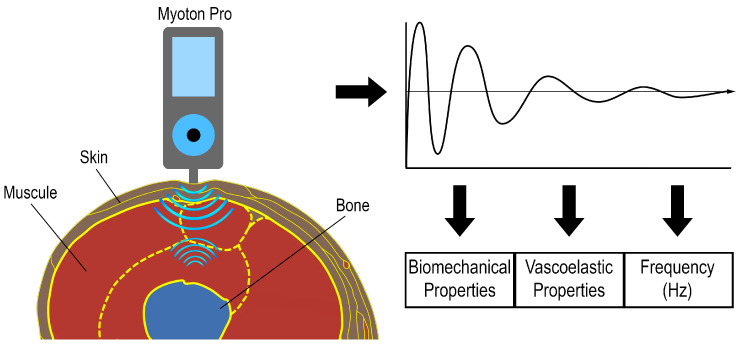
Diagram of myotonPRO work.

During the MyotonPRO-assisted examination, the probe placed perpendicularly on the examined area generated a series of electromagnetic oscillations and hit the examined muscle while the device collected data. It can be used to examine muscles, tendons, and fascia [34,35,36]. The examination assesses biomechanical properties (stiffness and deformation), viscoelastic properties (relaxation and creep), and muscle tension (frequency) [37]. These parameters are described in detail in Table 1 and Figure 1.

The present study aimed to achieve the following goals: (1) to find articles published before November 2024 in which the quality of GMED and TFL was assessed using MyotonPRO; (2) to check whether MyotonPRO has been used to examine muscles in patients with OA; (3) to identify potential changes in the activity of GMED and TFL induced by the development of HOA; and (4) to find articles in the available literature that assess the activity of these two abductors in patients treated with THA.

## 2. Materials and Methods

### 2.1. Literature Search: Databases and Keywords

The review of the international scientific literature was conducted by two independent experts who are specialists in the field of orthopedics and rehabilitation in October and November 2024. When necessary, a third reviewer made the final decision. The work consisted of searching Google Scholar, Science Direct, and PubMed databases. The following keywords were used to search the databases: osteoarthritis (OA), hip joint, abductor muscles, tensor fasciae latae (TFL), gluteus medius (GMED), MyotonPRO, muscle activity, lower extremity, and Total Hip Arthroplasty (THA). The keywords were entered separately or in combination. A literature review was performed according to the requirements outlined in the 2015 Preferred Reporting Items for Systematic Reviews and Meta-Analysis Protocols (PRISMA-P) statement (Supplementary Material—PRISMA 2020 Checklist).

### 2.2. Inclusion and Exclusion Criteria

The literature review included only articles published between 2012 and 2024. The research team established this time frame because earlier studies on myometry used other types of myometers than the MyotonPRO device. Only English-language papers that contained the keywords listed above and reported studies in which the MyotonPRO device was used to examine the GM and TFL in patients with OA were included in the review. Additionally, scientific studies presenting assessments of the activity and structure of the GM and TFL using other methods in patients with OA and in patients treated with THA were included in the review. The review included only studies conducted on patients aged 18 years and over. Papers written in languages other than English, articles published before 2012, studies that did not examine GM and TFL, and studies in a pediatric group (below 18 years) were excluded. Investigations conducted using myometers other than MyotonPRO for assessment of muscle quality were not included in the review.

### 2.3. Search Results

**Figure 2:**
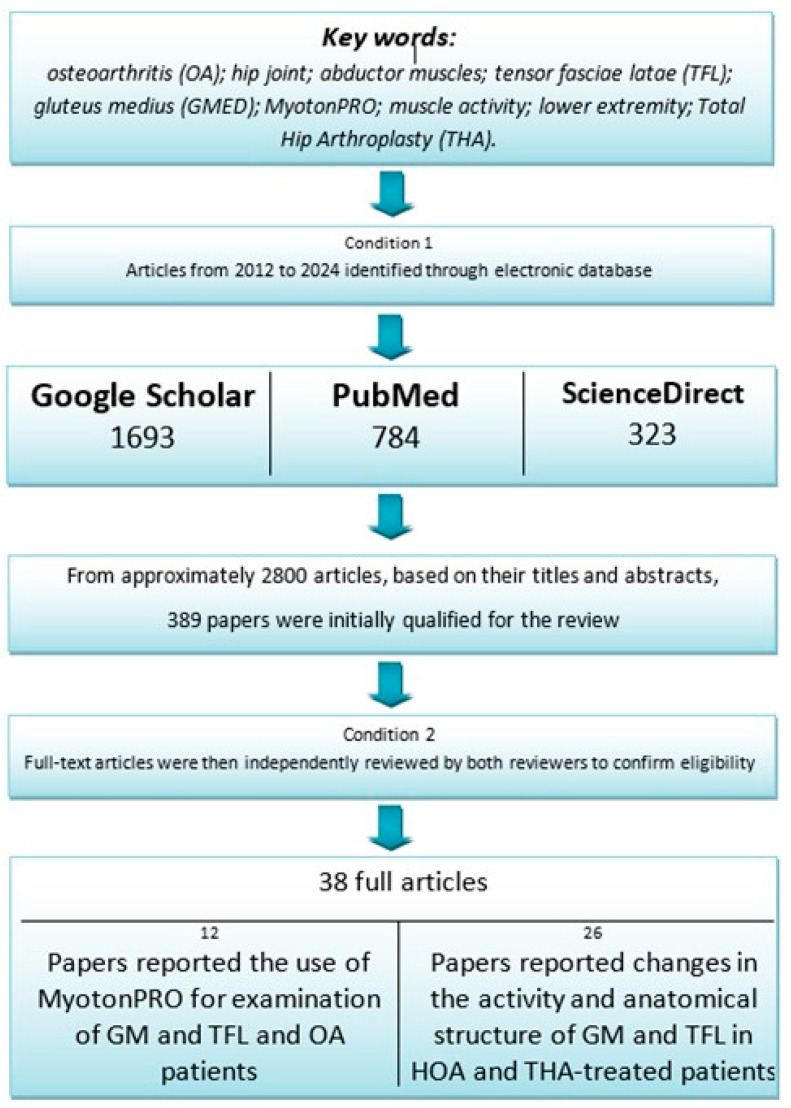
Diagram of the literature review scheme.

The database search performed using the keywords retrieved approximately 2800 articles. Based on their titles and abstracts, 389 papers were initially qualified for review. Two independent reviewers screened the titles and abstracts of all retrieved records to assess their eligibility based on predefined inclusion and exclusion criteria. Full-text articles were then independently reviewed by both reviewers to confirm their eligibility. Any discrepancies between the two reviewers at either stage were resolved through discussion or, if necessary, by consultation with a third reviewer. No automation tools were used during the selection process. Based on the inclusion and exclusion criteria specified above, 38 articles were included. Eleven papers reported the use of MyotonPRO for the examination of GM, TFL, and OA patients. Other studies focused on changes in GM and the activity and anatomical structure in patients with OA and THA-treated patients (Figure 2).

## 3. Results

### 3.1. Structure and Functions of GM and TFL

**Figure 3:**
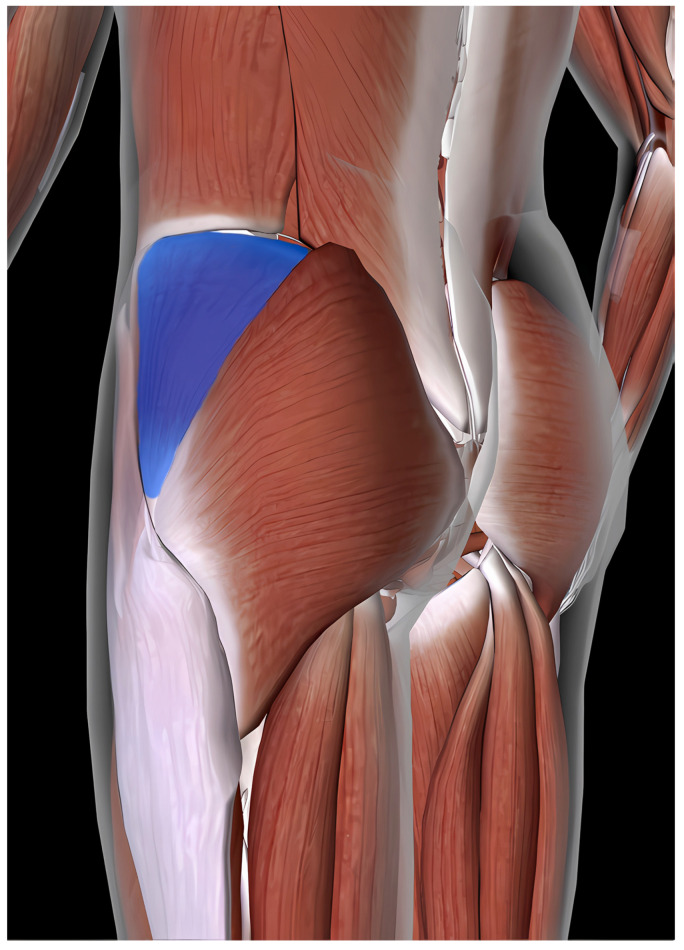
Gluteus medius (GMED) [40].

**Figure 4:**
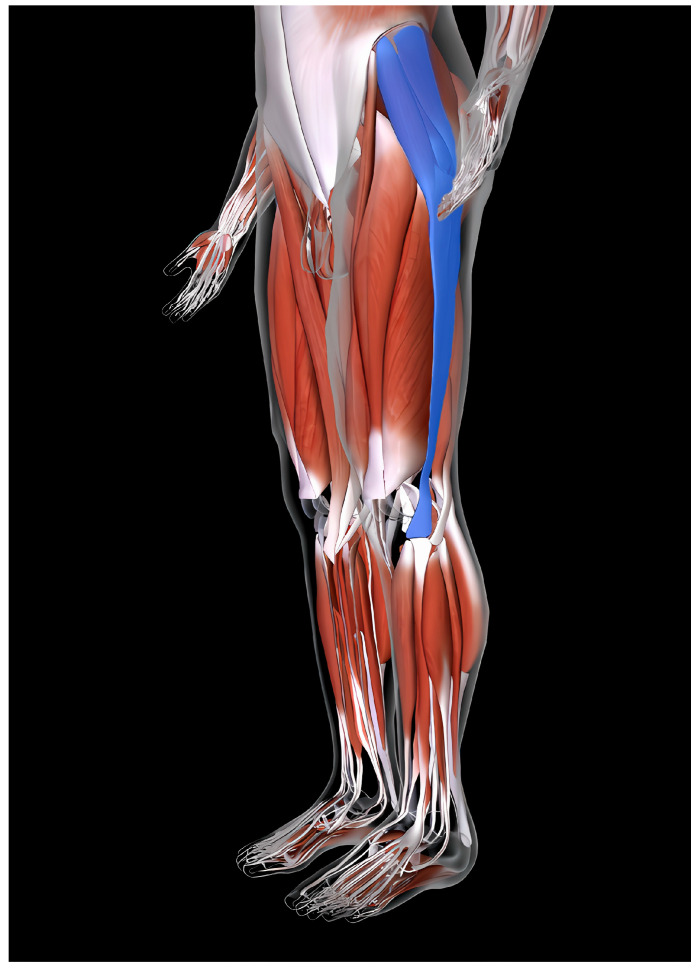
Tensor fasciae latae (TFL) [40].

The GMED and TFL muscles are the largest abductors. In terms of anatomical structure, the GMED begins on the gluteal surface of the iliac plate and lies between the upper and lower gluteal lines. It is located between the gluteus maximus and gluteus minimus. Its posterior part (1/3) is covered by the gluteus maximus muscle, while the superficial anterior part (2/3) is covered with a strong layer of deep fascia and is linked to the TFL. The posterior edge of the muscle runs along the upper edge of the piriformis and is separated from the muscle by gluteal vessels and nerves. The GMED ends with a short tendon on the greater femoral trochanter. Some of its fibers form the iliotibial band. Its structure exhibits a feathery arrangement of muscle fibers and a predominance of slow-twitch fibers [9]. The TFL begins with tendon fibers from the anterior superior iliac spine and gluteal fascia. The anterior part of TFL borders the sartorius muscle, and its posterior portion is closely linked to the GMED. Its outer surface borders the subcutaneous tissue, whereas the inner surface borders the GMED and the lateral head of the quadriceps femoris muscle. TFL fibers run parallel to the greater trochanter of the femur and form the iliotibial band of the fascia lata. The TFL ends below the lateral condyle of the tibia at Gerdy’s tubercle [38]. It has a flat, elongated, and quadrangular structure, which is characterized by a predominance of slow-twitch fibers, and is classified as a spindle-shaped muscle, similar to the GMED. The TFL and GMED muscles are innervated by the superior gluteal nerve (L4, L5) and vascularized by the superior gluteal artery [39] [Figure 3 and Figure 4].

GMED is the strongest hip abductor. During gait, it engages in alternating lateral movements of the pelvis. As a result, the pelvis tilts towards the loaded limb. The anterior part of the GMED in the hip joint is responsible for flexion and inward rotation, while the posterior part is involved in extension and outward rotation. GMED shortening and weakening are responsible for the appearance of the Trendelenburg sign [9]. Its weakening causes deterioration of pelvic stabilization, which can contribute to loss of balance and falls [13].

The TFL muscle interacts synergistically with gluteus medius, maximus, and minimus during internal rotation and abduction. Together with the iliopsoas muscle, it flexes the thigh at the hip joint. The fascia lata stabilizes the knee joint in extension, strengthening the vertical stance of the body. After exceeding knee flexion above 30 degrees, it serves as an additional knee flexor. Clinically, the basic function of the TFL is to support gait. It positions the hip bone downwards on the loaded side, thereby allowing the lifting of the other hip. Lifting the unloaded hip allows the lower extremity to perform the swing phase of the gait cycle. Shortening the TFL leads to anterior tilt of the pelvis and external rotation of the femur, whereas TFL lengthening causes the unloaded side to drop laterally, thereby disrupting the swing phase of gait [41,42].

Notably, the correct functioning of abductor muscles is influenced by pelvis positioning during gait, which depends on the correct biomechanics of the hip joint (e.g., femoral neck-shaft and femoral anteversion angles). Disturbed biomechanics of the hip joint contribute to the pelvis dropping on the unloaded side when the patient stands on the affected limb. This phenomenon is known as the Trendelenburg sign, and the patient’s gait is referred to as Trendelenburg gait, which is described as unsteady [39]. In symmetrical loading of both lower limbs while standing, the static load on the hip joint is approximately 1/3 of the body weight. In contrast, the static load on the hip joint while standing on one leg increases and constitutes as much as 250% of body weight. This is facilitated by the stabilization provided by the hip abductor muscles. The dynamic load on the hip joint may increase to 400% and 550% of body weight during walking and running, respectively. In the case of disease or damage to one of the hip joints, compensations that reduce the load emerge. The body’s center of gravity, which is normally located at the second sacral vertebra on the line connecting the hip joint axes, is shifted towards the axis of the damaged joint. This results in a decrease in the moment of gravity force and an increase in the angle of action of hip abductors [43].

### 3.2. GMED and TFL in Hip Osteoarthritis (HOA)

The activity and anatomical structure of the GMED and TFL muscles in patients with HOA were assessed in 12 studies [1,5,7,10,12,13,44,45,46,47,48,49]. The GMED was examined in all these studies [1,5,7,10,12,13,44,45,46,47,48,49], whereas only five studies focused on the examination of the TFL muscle [1,5,45,47,48] (Table 2).

#### 3.2.1. Assessment of GMED and TFL Activity in HOA

In five studies, GMED and TFL activities were assessed using EMG [10,12,13,44,45].

##### GMED Activity in HOA

An EMG study conducted by Zacharias et al., 2019 [44] revealed changes in GMED activity during gait. A significantly lower variability in the anterior hip GMED segments was recorded in the HOA group during the early and entire gait stance. However, the average or peak GMED activity in all parts (anterior, middle, and posterior) analyzed in the study did not differ significantly between the groups [44]. GM activity during gait was also assessed by Dwyer et al., 2013 [10], who observed that its amplitude was significantly higher during the stance and swing phases in the examined patients than in controls. In contrast, examination of the non-dominant limb showed significantly higher GMED activity in the HOA group only during the stance phase [10]. As reported by Zacharias et al., 2020 [13], the test of step-up, step-down, and side-step tasks indicated reduced activity of the middle and posterior GMED during the step-up and step-down task test in patients with HOA. Similarly, the anterior and posterior GMED during the side-step task exhibited later activation in the HOA group [13]. Different results for the same test were reported by Dwyer et al., 2013 [10]. Their study showed higher amplitude of GMED activity during the step-down and side-step tasks in limbs that were both involved and uninvolved in the movement in the HOA group [10]. Higher GMED activity, which is dependent on the severity of HOA, was also demonstrated by Rutherford et al., in 2015 [12]. The absence of changes in GMED activity on EMG examination compared to healthy controls was reported only by Schmidt et al., in 2016 [45]. The researchers observed no differences in the activity of this muscle between the HOA-affected and unaffected sides and found no differences in the asymmetry index (ASI) between the control and HOA groups [45] (Table 2).

##### TFL Activity in HOA

TFL activity was assessed according to Schmidt et al., 2016 [45]. In this study, TFL activity was higher in the HOA-affected limb than in the unaffected limb. Furthermore, there were no significant differences in ASI values between the HOA and control groups. However, the comparison of both limbs showed the highest TFL contribution to the total muscle activity, which was higher on the HOA-affected side. Differences in this parameter between the examined limbs were also found in the healthy group [45] (Table 2).

#### 3.2.2. Anatomical Structure of GMED and TFL

Seven studies considered in this review assessed the anatomical structure of the TFL and GMED muscles using MRI [1,5,7,47,48] or CT [46,49].

##### GMED Anatomical Structure in Patients with HOA

Zacharias et al., 2018 and Zacharias et al., 2016 observed GMED asymmetry in the HOA group [1,5]. Additionally, another study conducted by Zacharias et al., in 2018 showed that GMED asymmetry was correlated with HOA severity [1]. Regression analysis of the examination results obtained by Kawano et al., 2021 [46] showed that the cross-sectional area (CSA) and SMD (skeletal muscle density (SMD) of the GMED muscle were significantly correlated with hip abduction torque in the healthy limb. In contrast, no such relationship was observed in the HOA-affected limb [46]. Momose et al., 2017 [49] reported a correlation between CSA, GMED volume, and abduction force. Their study also showed a significant effect of the GMED muscle on hip abduction [49]. However, not all studies reported changes in GMED parameters [7,48]. For example, Loureiro et al., 2018 [48] showed no significant decrease in GMED volume [48]. The statistical analysis carried out by Peiris et al., 2020 [7] did not reveal any correlation between the CSA value and the quality of life of patients dependent on the hip joint function assessed with the HOOS (Hip Disability and Osteoarthritis Outcome Score) [7] (Table 2).

##### TFL Anatomical Structure in Patients with HOA

No changes in the TFL volume and symmetry were demonstrated by Zacharias et al., 2018, Zacharias et al., 2016, and Loureiro et al., 2018 [1,5,48]. In a study conducted by Homma et al., (2023), the TFL muscle exhibited more advanced fatty degeneration than the GMAX. The authors also reported a correlation between the TFL volume and its CSA. However, statistical analysis showed that TFL strength was not significantly correlated with CSA [47] (Table 2).

### 3.3. GMED and TFL Activity and Anatomical Structure in THA-Treated Patients

The activity and anatomical structure of the GMED and TFL were assessed in 13 studies on THA-treated patients [50,51,52,53,54,55,56,57,58,59,60,61,62]. GMED was examined in all these studies [50,51,52,53,54,55,56,57,58,59,60,61,62], whereas TFL was examined in five publications [52,54,56,58,59] (Table 3).

#### 3.3.1. GMED and TFL Activity

GMED and TFL activities were assessed in four publications included in this review; the muscles were examined using EMG in three studies [52,56,58] and with shear wave elastography (SWE) in one study [54] (Table 3).

##### GMED and TFL Examined with the Use of EMG

In a study conducted by Bernard et al., 2018 [52], no differences in GMED and TFL activity were noted between examinations performed at a 1-week interval. The researchers also found that the GMED muscle exhibited higher activity during the unipodal and bipodal balance tests on both the operated and non-operated sides compared to healthy controls. Body posture disorders have also been observed [52]. Khalif et al., 2023 [58] used EMG to assess GMED and TFL activity, and motor unit action potentials (MUAPs) were calculated based on the results of the examination. In the study group, denervation of the abductor muscles was observed in 15 patients at 6 weeks post-THA. After 12 weeks, denervation persisted in seven patients in this group, while spontaneous regression of the changes was observed in eight patients. The MUAP values after 12 weeks were slightly different from those calculated prior to THA, which indicated restoration of normal muscle activity [58]. In a study conducted by Horstmann et al., 2013 [56], EMG was performed 6 months before and after the procedure. The results were compared with those obtained in healthy controls. The analysis of the results revealed that the motion ranges, activity, and duration and distance of gait at 6 months post THA differed from the preoperative levels and were similar to the results recorded in the examination of the contralateral limb and the healthy control. The researchers also described changes in TFL and GMED activities during different gait phases. The TFL muscle exhibited an additional EMG intensity peak at the end of the stance phase in patients with THA before and after surgery compared to healthy controls. In turn, higher EMG results were obtained for GMED only in the initial gait phase [56] (Table 3).

##### GMED and TFL Examined with the Use of SWE

Kinoshita et al., 2021 [54] conducted the only study where GM and TFL were examined using SWE. In this study, THA-treated patients were divided into two groups. The division criterion was the patient’s perception of a difference in the length of their lower limbs (PLLD—perceived leg length discrepancy). Statistical analysis of the results showed higher elasticity of abductor muscles before THA in the group of patients with PLLD. Similarly, regression analysis showed that the size of the preoperative elastic modulus impacted PLLD [54] (Table 3).

#### 3.3.2. GMED and TFL Anatomical Structure

Nine studies assessed the anatomical TFL and GMED structures in THA-treated patients [47,48,50,52,54,56,57,58,59]. MRI was performed in three studies [51,53,57,61], while CT was used by six research teams [50,55,57,59,60,62] (Table 3).

##### GMED Anatomical Structure

As reported by Yasuda et al., 2022, changes in the anatomical GMED structure may impact postoperative outcomes in patients who underwent THA [50]. Similarly, Hu et al., 2020 [51] observed that the GMED structure may affect postoperative performance. GM shortening may negatively affect post-THA joint stability [51]. A reduction in GMED length was also reported by Manafi Rasi et al., 2020 [53]. However, patients in their study exhibited improved functional outcomes [53]. A reduction in GMED volume was also observed by Nankaku et al., 2016 [55]. Their study showed that changes in the GMED muscle may cause limping that may persist for up to 6 months post-THA [55]. Various relationships between some CT-assessed GMED parameters and gait speed after THA were observed by Yasuda et al., 2023 [57]. In their study, the LMM/TM (lean muscle mass/total muscle mass) parameters were negatively correlated with postoperative gait speed in the entire THA group. In contrast, the LDL/TM (low-density lean tissue/total muscle area) indicator was positively correlated with postoperative improvement in gait speed. A positive relationship between the amount of LDL (low-density lean tissue) and postoperative results was also found. In terms of sex groups, a positive relationship between LMM and LMM/TM with postoperative improvement in gait speed was found in the female group [57]. An increase in the CSA of the GMED at 2 years post-THA was reported by Uemura et al., in 2016 [62]. Their study also demonstrated that the increase in the CSA of this muscle was associated with improvement in functional test results in the examined group of patients [62] (Table 3).

In a study conducted by Kawakami et al., 2024 [60], THA-treated patients were divided into two groups based on the results of the TUG (Timed Up and Go) test. The strength and quality of the healthy limb GMED, but not the THA-treated side, had an important impact on the TUG test conducted one year after surgery [60]. As shown by Kovalak et al., 2018 [61], considerable fatty degeneration of the GMED was observed on the operated side compared to the non-operated limb. The statistical analysis conducted in their study revealed a negative effect of steatosis on HHS results. The results of this study showed a relationship between muscle steatosis and the age of patients [61]. Damm et al., 2018 [59] analyzed the anatomical structure of GMED before and 3 months after the procedure. Their analysis showed an insignificant decrease in the lean muscle volume and an insignificant increase in the degree of fatty degeneration. Additionally, correlation analysis revealed a significant effect of fatty degeneration on hip joint contact forces during sitting, standing, and walking [59] (Table 3).

##### TFL Anatomical Structure

The TFL anatomical structure was assessed using CT only by Damm et al., 2018 [59]. A non-significant increase in total volume and an increase in lean muscle volume were observed. Statistical analysis showed a significant decrease in the level of fatty degeneration of this muscle. As in the case of the GM muscle, the correlation analysis revealed a significant effect of fatty degeneration on hip joint contact forces during walking [59] (Table 3).

### 3.4. MyotonPRO-Assisted Examinations

#### 3.4.1. Assessment of GMED and TFL

Only five studies in the currently available literature have used a MyotonPRO device to examine GMED and TFL muscles [63,64,65,66,67]. Two studies examined GM stiffness and flexibility [63,64], and TFL was assessed in four studies [61,62,63,64] (Table 4).

Two of these studies assessed the stiffness and flexibility of GMED and TFL muscles in patients with LBP (low back pain) and CPP (chronic pelvic pain) [63,64]. Kim et al., 2022 [63] compared two types of exercises and their effect in LBP treatment. Regardless of exercise type, lower GMED stiffness was noted in both groups before the start of the rehabilitation program [63]. In contrast, Proulx et al., 2023 [64] compared two groups with CPP with an asymptomatic group. No significant differences in TFL and GMED stiffness were observed between the groups [64] (Table 4).

The other three studies qualified for this review focused on examinations of athletes [65,66,67]. The researchers found that the biomechanical properties of muscles and resting tension did not change after long-term exercise [67]. As reported by Wolska et al., 2023 [66], the use of cryosauna after intense exercise did not reduce TFL stiffness induced by delayed onset muscle soreness (DOMS) [66]. In turn, Mroczek et al., 2019 [65] reported a statistically insignificant increase in muscle stiffness as a result of PT (plyometric training), which nonetheless improved CMJ (countermovement jump) [65] (Table 4).

#### 3.4.2. Examination of Muscles in the OA-Affected Lower Limb

Only six publications included in this review reported examinations using MyotonPRO to assess lower limb muscles in patients with OA. These examinations involved only patients with knee osteoarthritis (KOA) [14,15,16,17,68,69] (Table 5).

The following muscles were examined in these studies: rectus femoris (RF), vastus lateralis (VL), vastus medialis (VM), biceps femoris (BF), gastrocnemius medialis (GM), and gastrocnemius lateralis (GL). The RF muscle was examined most frequently and was assessed in five papers [14,16,17,68,69]. VL and VM were assessed by three research teams [14,16,17]. In contrast, BF, GM, and GL were examined in a single study [15,68] (Table 5).

MyotonPRO-assisted analyses have revealed changes in the biomechanical properties induced by the development of KOA in these muscles [14,15,16,17,68,69]. In three of the six articles considered in this review, the MyotonPRO results of patients with KOA were compared with those of healthy controls [14,17,69]. These investigations demonstrated significantly higher stiffness and tension of the VL in patients with KOA than in healthy controls [17]. Similarly, Chang et al., 2021 [14] reported an increase in the stiffness of this muscle in the KOA group compared to healthy controls. Their statistical analysis did not reveal any changes in stiffness in the other muscles examined in their study (RF and VM). The researchers showed that muscle stiffness correlated with the degree of disability. Their study also revealed that the stiffness of the examined muscles increased with age, regardless of the group, that is, both in patients with KOA and in healthy volunteers [14]. In a study conducted by Ohko et al., 2020 [69], patients with KOA were compared to healthy controls. A decrease in elasticity has been shown to impact patella mobility, which is significantly correlated with OA [69] (Table 5).

Shen et al., 2024 [16] compared KOA-affected and unaffected sides in over 50-year-old females. The affected side exhibits higher tension and stiffness of the quadriceps muscle than the unaffected limb [16]. The same comparison was made in the study conducted by Li et al., (2024) [17]. The researchers found asymmetry in the biomechanical parameters of the quadriceps femoris muscle between the affected and unaffected sides in patients with unilateral KOA. In the KOA group, RF and VM tension on the affected side was significantly higher than that on the healthy side. In turn, the VL stiffness on the KOA-affected side was significantly higher than that on the unaffected limb [17] (Table 5).

Different group assignments were employed by Chen et al., 2021 [15]. In their study, the examination results of patients with unilateral KOA were compared with those obtained in a group with bilateral changes. A significantly higher degree of GL stiffness was diagnosed in limbs with more advanced degenerative changes. A correlation was observed between the biomechanical properties of this muscle and the severity of arthrosis [15] (Table 5).

The relationship between the biomechanical properties of RF and BF and impaired joint loading was investigated only by Murillo et al., 2023 [68]. Their study showed that impaired BF affects hip joint loading and thus exerts a negative effect on the intensity of pain experienced by patients [68] (Table 5).

## 4. Discussion

Reduced muscle mass, also referred to as muscle atrophy (sarcopenia), is not only associated with aging but also accompanies various diseases (e.g., HOA), causing limitations in everyday functioning. HOA is associated with pain, muscle weakness, and disuse of the affected limb, leading to limited physical performance. Muscle atrophy observed in this group of patients was associated with inactivity. Additionally, over 31% of patients with HOA exhibit reduced muscle strength [70]. Notably, some studies suggest that weakening of the muscles surrounding joints affected by degenerative changes may further support the development of this disease [5].

Hip abductors, such as GMED and TFL, are among the most important muscles influencing the biomechanics of the lower extremity [5,7]. These muscles not only play a key role in hip abduction but also have an important effect on pelvic stabilization and proper gait. Disturbances in hip abduction may cause pain, thereby limiting hip joint function [8,71]. Elucidating the function of abductor muscles and the impact of their weakness on the functioning of patients with HOA is essential for designing rehabilitation programs [8].

The aim of this literature review was to collect information about the possibility of using the MyotonPRO device to assess the quality of the abductor muscles, in particular GMED and TFL, in HOA-affected and THA-treated patients. Another goal was to determine whether the available literature indicates that the proposed research trend is innovative, novel, and feasible. Hence, the review included studies on the assessment of GMED and TFL using other methods in HOA-affected and THA-treated patients. Additionally, the review aimed to determine whether MyotonPRO was used to examine these muscles and patients with degenerative joint disease.

The present review, performed with consideration of the inclusion and exclusion criteria specified in the Materials and Methods section, showed that 12 studies assessed the activity and anatomical structure of the GMED and TFL in patients with osteoarthritis [1,2,5,7,9,10,41,42,43,44,45,46]. GMED was examined in all these studies [1,5,7,10,12,13,44,45,46,47,48,49], whereas only five research teams examined the TFL muscle [1,5,45,47,48].

The activity of the GMED and TFL muscles was assessed using EMG in five studies [10,12,13,44,45]. Their results were compared to those of a healthy control by five research teams [10,13,44,45], and GMED activity was evaluated in relation to the degree of degeneration in the hip joint in one study [12]. In a study conducted by Dwyer et al., 2013 [10], patients with OA exhibited increased GMED activity during gait in the stance and swing phases in the dominant limb and only in the stance phase in the non-dominant extremity. In the same study, the step-down and side-step tests in the OA group revealed increased activity of this muscle in both limbs, regardless of which limb was involved in the movement [10]. Rutherford et al., 2015 [12] did not compare patients with HOA to healthy controls; however, the patients were divided into three groups according to the severity of the disease. GMED activity increases with the degree of degeneration [12]. Contrasting results, i.e., a decrease in GMED activity, were reported in two studies [13,44]. The statistical analysis of the results collected by Zacharias et al., 2019 [44] in the HOA group showed a significantly lower variability in the anterior hip segments of GMED in early and entire gait stance. In turn, the average or peak GMED muscle activity of all the examined GMED parts did not differ significantly between the studied groups [44]. A study published by the same research team a year later showed a significant decrease in the results of EMG examination of the GMED. During the step-up, step-down, and side-step tasks, the group of patients with HOA exhibited reduced activity of the middle and posterior GMED during the step-up and step-down tasks. Similarly, the anterior and posterior GMED parts were characterized by delayed activation during the side-step task in the OA group [13]. The EMG examination did not show changes in GMED activity only in the study carried out by Schmidt et al., 2016 [45]. In their study, there were no differences in the activity of this muscle between the HOA-affected and-unaffected sides. No changes in the asymmetry index (ASI) were observed between the control and HOA groups [45]. As mentioned earlier, TFL was examined by Schmidt et al., (2016) [45]. In this study, TFL activity was higher in the HOA-affected limb than in the unaffected limb [45].

Contradictory results were reported in the analyzed articles, especially in the two publications by Zacharias et al., 2019 [44] and Zacharias et al., 2020 [13], in which no increase in GMED activity was observed in patients with HOA [13,44]. Statistical analysis of the results showed a significant decrease in the values obtained during the EMG examination of this muscle. The differences in the results may be related to the fact that Zacharias et al., 2019 [44] and Zacharias et al., 2020 [13] used needle EMG to assess GMED activity, while surface EMG was employed in the other studies [10,12,45]. Needle EMG is an invasive examination; therefore, it is less frequently used in practice. Insertion of needle electrodes requires experience, and the examination process is long [72,73,74]. Nevertheless, the use of this type of electrode in Zacharias et al., 2019 and Zacharias et al., 2020 allowed the GMED to be divided into three segments (anterior, middle, and posterior); hence, the differences between the analyzed studies [13,44]. The use of needle electrodes minimizes signal interference, which may also impact the examination results [72,73,74]. Research has shown that the thickness of subcutaneous tissue may influence the examination results provided by EMG with self-adhesive electrodes [72,75,76]. In the studies analyzed in this review, the thickness of subcutaneous tissue on the examined muscles was not measured, and the correlation between its thickness and the activity of the muscles was not determined [10,12,45]. Additionally, no studies have compared the use of surface and needle EMG techniques for the assessment of GMED and TFL activity in patients with HOA. These findings indicate that the use of different activity assessment methods may impact examination results. Therefore, this issue requires further investigation.

Notably, Zacharias et al., 2019 [44] and Dwyer et al., 2013 [10] used different criteria for gait assessment. Step-down and side-step tests were performed in both studies, but their results differed, which may have been related to the age of the patients. The average age of patients was 50 years in the study conducted by Dwyer et al., 2013 and 70 years in the analyses reported by Zacharias et al., 2019 [10,44]. Research has shown that reduced muscle activity in elderly individuals is associated with reduced physical performance and progressive steatosis of muscle tissue [77,78].

The present review analyzes studies assessing the relationships between GMED and TFL activity and their anatomical structures. The anatomical structures of these two muscles have been investigated in seven publications [1,5,7,46,47,48,49]. Some of the papers have reported changes in the GMED muscle [1,5,46,49]. GMED asymmetry was demonstrated in the HOA group, and it was found that the asymmetry was correlated with the severity of HOA [1,5]. Regression analysis performed in other studies indicated that CSA and SMD values were significantly correlated with the abduction range in the healthy limb in patients with HOA. In contrast, no such relationship was found in HOA-affected limbs [46]. Momose et al., 2017 found a relationship between the CSA and the GM volume, abduction force, and the effect of GMED on hip joint abduction [49]. Peiris et al., 2020 and Loureiro et al., 2018 did not report any changes in GMED [7,48]. The statistical analysis of the results obtained by Loureiro et al., 2018 revealed no changes in GMED volume [48]. The correlation analysis performed by Peiris et al., 2020 did not confirm the relationship between the CSA of the GMED and hip joint function assessed using the HOOS scale [7]. In the case of the TFL, imaging by Homma et al., 2023 [47] showed a relationship between its volume and CSA value. However, no correlation has been observed between the strength and CSA of this muscle [47]. Zacharias et al., 2018, Zacharias et al., 2016, and Loureiro et al., 2018 found no changes in the TFL volume and asymmetry [1,5,48].

The differences between the results of the studies described in this review may be related to the relatively small number of patients with HOA examined, which may have had a significant impact on the results [1,5,7,46,47,48,49]. Additionally, in some studies, the examination results were not compared with those of healthy or asymptomatic controls. The volume, asymmetry, and CSA results were compared to those of the control group only by Zacharias et al., 2018, Zacharias et al., 2016, and Loureiro et al., 2018 [1,5,48]. The analyses performed in other studies consisted of determining the relationship between the analyzed parameters, which limited the comparison of the results [7,46,47,49]. Potential measurement errors should also be taken into account, as the muscle parameters may have been measured randomly in different areas and at different depths, which may also have a significant effect on the results. Additionally, the results may have been influenced by the experience of the examiner, as well as the calibration and type of device used to perform the imaging examination. These factors may have affected the determination of the anatomical borders of the analyzed muscles [7,79,80,81]. The type of tool used in the study may also have impacted the results [79,82]. These findings indicate that the use of different methods may impact the examination results. In addition, given the multitude of factors that may impact the reliability and repeatability of the results, further research in this field is recommended.

The differences in the results of the studies discussed may also be due to the differences in the study groups in terms of age, sex, and size. The limitations of the results may also stem from the lack of a healthy control group. The statistical tests used may have also influenced the results obtained in the studies discussed [83].

Noteworthy is the fact that the growing number of HOA patients and the increasing number of THA procedures suggest the need to conduct comprehensive studies on the etiology, development, and treatment of this disease [8]. Currently, the treatment of HOA is limited, and only symptomatic therapy is available; surgical treatment is possible in advanced cases [6]. According to epidemiological data, the frequency of THA is expected to increase by up to 176% by 2040 [19].

THA outcomes are satisfactory, as over 90% of patients report excellent or good results post-surgery [84,85]. However, a simultaneous decrease in muscle strength, most often in the abductors, has been observed in some THA-treated patients [84,86,87]. Research has shown that the physical performance of patients after THA is largely associated with the proper functioning of the abductor muscles. They have a significant impact on the occurrence of limping in THA-treated patients [84]. Abductor atrophy after THA was diagnosed in 22% of patients, regardless of the surgical technique used. However, it should be emphasized that the dysfunction of abductors post THA is a completely different clinical entity from their preoperative atrophy. THA-postoperative abductor deficiency can be caused by intraoperative damage, muscle atrophy resulting from superior gluteal nerve damage, failure to repair already damaged muscles, rupture of the abductor tendon attachment, or implant loosening. Regardless of the cause, the diagnosis and therapy of abductor muscle dysfunction are difficult and may result in unsatisfactory functional outcomes in patients undergoing THA [71].

The present review included studies that assessed the activity and anatomical structure of the GMED and TFL muscles in patients who underwent THA. The inclusion and exclusion criteria established in the review qualified 13 papers [50,51,52,53,54,55,56,57,58,59,60,61,62]. GMED was analyzed in all publications [50,51,52,53,54,55,56,57,58,59,60,61,62], whereas TFL was examined by five research teams [52,54,56,58,59].

GMED and TFL activity, as well as flexibility, were assessed using EMG and SWE in four studies [52,54,56,58]. In two of the four papers, the results were compared not only to the results obtained prior to the surgery but also to the healthy asymptomatic control [52,56]. The analysis of GMED and TFL activity performed by Horstmann et al., 2013 showed that, at 6 months post THA, the activity of the examined muscles did not differ from that assessed in the HOA-unaffected limb and in the healthy control, which may indicate that the surgery restored the normal activity of the muscles [56]. In contrast, Bernard et al., 2018 described balance disorders and higher GM activity during the balance test (Unipodal and Bipodal) [52]. The differences between these studies were not associated with the surgical approach, as THA was performed using the lateral approach in all patients [52,56]. However, the differences between these studies may be related to the different time points of post-surgery muscle assessment. The EMG examination presented by Bernard et al., 2018 [52] was performed 45–60 days after the procedure, whereas Horstmann et al., 2013 [56] assessed the activity of abductor muscles 6 months after surgery. This may have had a significant impact on the results, as the examination of the muscles was carried out in different phases of healing [52,56,88,89,90,91,92]. The period between 21 and 60 days post-surgery is the healing phase III, i.e., the consolidation phase, characterized by an increase in muscle mass. Muscles are then able to handle greater loads, which may contribute to the increased GM activity observed during examination. The muscle is also in the process of regaining its mass, which may lead to balance disorders. The completion of phase III is followed by phase IV, i.e., remodeling, which takes up to a year [88,89,90,91,92]. The connective tissue healing process is completed during this phase, and the muscle regains its appearance and mass, which may have been observed in patients by Horstmann et al., in 2013 [56]. Similarly, Khalif et al., 2023 [58] reported slightly different EMG examination results in patients at 12 weeks post-surgery compared to the results obtained prior to THA, which may indicate restoration of the abductor function. In the same study, abductor denervation was observed in 15 patients at 6 weeks post-THA. However, THA was performed using the anterior approach in the patients analyzed in this study. This may have been responsible for the damage to the muscles and nerves, which regenerated during the convalescence and rehabilitation processes [52,56,58,88,89,90,91,92]. Recent studies have shown that the anterior approach to the hip joint may cause damage to abductor muscles and to the lateral cutaneous nerve of the thigh, which may explain the muscle denervation reported by Khalif et al., 2023 [58,93,94,95]. Surface EMG was used for examination by Bernard et al., 2018 [52] and Horstmann et al., 2013 [56], while Khalif et al., 2023 [58] used needle EMG, which showed changes in the activity of the analyzed muscles more accurately [52,56,58,72,73,74].

In turn, a different division of patients was used by Kinoshita et al., 2021 [54]. They were divided into two groups according to PLLD, and the properties of muscles were assessed using SWE. Statistical analysis of the examination results showed that changes in elasticity impact PLLD post THA [54].

The present review also included studies that assessed the anatomical structure of GMED and TFL muscles in THA-treated patients [50,51,53,56,57,59,60,61,62]. As mentioned above, structural changes have a significant impact on muscle activity and function [77,78]. As reported in the analyzed publications, changes in the GMED structure may affect postoperative outcomes in patients who underwent THA [50,51]. Hu et al., 2020 demonstrated that shortening of the GMED length may exert a negative effect on joint stability after total hip arthroplasty [51]. Shortening of this muscle has also been reported by Manafi Rasi et al., 2020 and Nankaku et al., 2016 [53,55]. Concurrently, Manafi Rasi et al., 2020 found improved functional test results in THA-treated patients [53]. In turn, Nankaku et al., 2016 observed that GMED shortening may cause limping in patients at 6 months post THA [55]. A correlation analysis performed by Yasuda et al., 2023 [57] revealed negative and positive relationships between some GMED parameters and postoperative gait speed. Their study showed a negative correlation between LMM/TM and gait speed in patients who underwent THA, whereas the relationship between LDL/TM and LDL with patient outcomes after surgery was positive. These results suggest that an increase in lean muscle mass and its ratio to total muscle mass contributes to better functioning in THA-treated patients [57]. Similarly, Uemura et al., 2016 [62] found that an increase in GMEDCSA was associated with improved functional outcomes post-THA [62]. Statistical analysis performed by Kawakami et al., 2024 revealed a negative impact of fatty degeneration on HHS outcomes, which emphasizes the importance of an increase in lean muscle mass [60]. Fatty degeneration of both GMED and TFL was also examined by Damm et al., 2018 [59]. Statistical analysis showed an increase in fatty degeneration, which was insignificant for GMED but significant for TFL. Correlation analysis indicated that GMED fatty degeneration had a significant effect on hip joint contact force during activities such as sitting, standing, and walking. It was also demonstrated that TFL fatty degeneration had a significant impact on hip joint contact force during walking [59].

It should be noted that the results of the imaging-assisted assessment of the GMED and TFL performed before and after THA were not compared with healthy controls in any of the analyzed studies [50,51,53,55,57,59,60,61,62]. As mentioned before, the small number of examined patients in all studies may have influenced the results, as well as the differences in the imaging and analysis techniques used, which were discussed earlier in greater detail [7,79,80,81,82]. The surgical approach used during THA also impacted the GMED and TFL structure assessment results. In the studies included in this review, the THA procedure was performed using the lateral approach in four studies [50,56,57,59], posterolateral approach in two studies [61,62], anterolateral approach in one study [55], anterior approach in one study [60], and posterior approach in one study [51]. In two studies, THA was performed using the Hardinge modification [53] and the posterolateral modified Watson-Jones approach [60]. Each of these approaches has its own advantages and disadvantages. Different surgical techniques are employed, and different muscles are cut (or not) in each approach [18,96,97]. Therefore, further research in this field is highly recommended.

The present review of the publications cited above highlights the importance of the GMED and TFL in HOA-affected and THA-treated patients. These muscles have been shown to markedly influence patient performance. Changes in their structure and activity may impact postoperative outcomes.

In addition, the present review has shown that the GMED muscle has been studied most frequently in patients with HOA and after THA [1,5,7,10,12,13,44,45,46,47,48,49,50,51,52,53,54,55,56,57,58,59,60,61,62]. The TFL muscle has been examined less frequently in patients with HOA and after THA [1,5,46,47,48,54,56,58,59], which may be related to its location and the difficulty in its assessment [38,39]. As mentioned earlier, both muscles play key roles in the proper functioning of the hip joint [5,7,8].

A unique aspect of this review is the finding that no studies have assessed the biomechanical and viscoelastic properties of the GMED and TFL using the MyotonPRO device in HOA-affected and THA-treated patients. The review showed that this device was used for the examination of GMED and TFL muscles, but in a different group of patients. Two studies assessed the stiffness and elasticity of replaced muscles in patients with LBP and CPP [63,64]. Three other studies assessed the muscles of athletes [65,66,67]. Two studies examined GMED stiffness and flexibility [63,64], and four studies assessed these parameters in TFL muscles [64,65,66,67]. The present literature review showed that lower limb muscles were assessed in patients with OA using MyotonPRO in six publications. However, these studies included only patients with KOA [14,15,16,17,68,69]. The researchers examined other muscles, including the RF, VL, VM, BF, GM, and GL. Their studies showed changes in the biomechanical properties of the examined muscles during the development of KOA [14,15,16,17,68,69]. These studies indicate that myotonPRO can be used to assess lower limb muscles. However, there are limitations due to the small number of studies. It should also be noted that these studies involved a variety of patient populations in terms of health status, physical fitness, sex, and sample size. Notably, a certain number of studies that were not considered in this review due to the specified inclusion and exclusion criteria showed that the results of the assessment of both lower and upper limb muscles are reliable and repeatable [28,29,33,36,37,98,99,100]. The results obtained during the examination using myotonPRO have been shown to be reliable and repeatable. Reliability has been demonstrated in the assessment of the gastrocnemius muscle [24], quadriceps [15,25], and tendons in tendinopathies [27]. This device has also been used to assess changes in muscles resulting from paratonia in dementia [29] and in Parkinson’s disease [30]. Studies have also confirmed the clinical value of MyotonPRO, demonstrating its usefulness in the assessment and monitoring of treatment in patients with stroke [33].

In addition, many researchers have highlighted the advantages of using MyotonPRO. Compared to other methods for analyzing the biomechanical properties of muscle tissue, such as needle/surface EMG or elastography, the MyotonPRO device is easy to use, does not require special laboratory conditions, is non-invasive, and is cheaper to use. The operation of this device only requires appropriate examiner training and identification of correct landmarks [28,29,33,36,37,98,99,100]. Therefore, further research is required in this regard.

To our knowledge, studies using MyotonPRO in patients after THA should be conducted at times consistent with tissue healing phases. Tissue healing progresses through four phases: acute (I), proliferation (II), consolidation (III), and remodeling (IV). Due to its hemodynamic instability, Phase I should not be considered. In Phase II, its peak transformation occurs between weeks 2 and 3; during this period, inflammation decreases, and collagen synthesis increases. In the subsequent Phase III, lasting 21–60 days, collagen fiber hypertrophy occurs, which contributes to an increase in the cross-sectional area of the striated muscles, resulting in increased muscle function. In the final Phase IV, lasting up to a year, the healing process is completed, and muscle fibers regain full functional capacity [88,89,90,91,92]. The MyotonPRO study will demonstrate the changes that occur during GMED and TFL healing following THA. In patients with HOA, studies using this device should be conducted at various stages of HOA development, and a properly selected healthy control should be used to demonstrate changes in GMED and TFL due to the development of this disease entity. In addition, personnel performing this examination must be properly trained. The probe application points should also be consistent for all patients. It is important to note that, like any other device, the MyotonPRO has its limitations. This examination requires appropriate training and predetermined measurement points. It is important to note that this examination is performed percutaneously, and its results can be significantly affected by the thickness of the subcutaneous layer [101]. Therefore, we believe that examinations using MyotonPRO should be supplemented with imaging tests, such as ultrasound, to assess the thickness of the subcutaneous tissue. This will allow for the assessment of the correlation between the results obtained in the MyotonPRO examination and the thickness of the subcutaneous layer. Furthermore, examinations in which MyotonPRO is used should be supplemented with EMG studies, which also assess changes in muscle biomechanics, contributing to a more detailed assessment of the muscles [72,73].

The limitations of this review include the inclusion and exclusion criteria. One limitation of our review is that it was limited to studies written exclusively in English, excluding potentially relevant publications in other languages. Another limitation of our work is the time scale of publication of the articles, i.e., only publications issued since 2012 were considered. Nevertheless, the time limit allowed for the exclusion of studies in which myometry was performed using other types of myotonometers. In order to standardize the review, the established time frame was also applied to publications in which GMED and TFL activity in HOA-affected and THA-treated patients was examined using other diagnostic methods. Another limitation factor is the lack of a meta-analysis as well as the lack of an automated literature search tool, which could have improved the selection process and increased the transparency, reproducibility, and consistency of studies. However, in our study, to exclude bias, the literature review was conducted by two independent experts in orthopedics and rehabilitation, and a third expert was consulted in case of any controversial issues.

Despite these limitations, this review highlights the importance of the GMED and TFL muscles in hip osteoarthritis and their impact on return to full function in THA-treated patients. These studies have reported contradictory outcomes in both OA-affected and THA-treated patients. Many factors that may influence these outcomes are also discussed. This is the first review article to show that no studies have reported the use of MyotonPRO in this group of patients. Concurrently, this study indicates a new direction of research aimed at testing the possibility of using MyotonPRO to examine GMED and TFL in knee and hip osteoarthritis. The literature presented in this review has also shown that the use of MyotonPRO can be a very good method for muscle assessment. Its results are repeatable and reliable, and the examination is non-invasive and easy to perform; hence, it is a very efficient method for assessing lower limb muscles. However, there are limitations due to the small number of studies. Therefore, further research in this field is warranted.

## 5. Conclusions

The information provided by the reviewed articles can contribute to the elucidation of GMED and TFL functions and their impact on both the performance of patients with HOA and the development of hip osteoarthritis. Research conducted in this field will reveal a potential effect of THA on the restoration of normal activity of abductor muscles and proper functioning of the hip joint. It should also be emphasized that no studies have assessed the biomechanical and viscoelastic properties of GMED and TFL using the MyotonPRO device in HOA-affected and THA-treated patients. Nevertheless, our work has indicated that MyotonPRO can be a valuable tool for assessing changes in GMED and TFL in various groups of patients and assessing other muscles in patients with KOA. However, it should be noted that studies using MyotonPRO are new and require further research focused on how to perform the examination and which muscles should be tested. It is also necessary to determine the time after THA when the MyotonPRO examination can be performed so that the results obtained during this examination will have a significant impact on the treatment of these patients. Extensive investigations of the use of MyotonPRO may help support the more precise development of rehabilitation programs for HOA-affected and THA-treated patients. The results of this research may help to delay the progression of osteoarthritis and promote a faster return to full functionality in patients undergoing total hip replacement.

## Figures and Tables

**Table 1:** Parameters assessed by MyotonPRO [37].

MyotonPRO	
Tension	
Frequency	[Hz] natural oscillation characterizing intracellular muscle tension
Biomechanical properties	
Stiffness	[N/m] tissue resistance to contraction or to the action of applied force
Deformation/elasticity	[-] tissue’s ability to return to its original state after cessation of the action of force or cessation of contraction
Viscoelastic properties	
Relaxation	[ms] time between the maximum deformation of muscle tissue and its return to the initial state
Creep	[-] gradual elongation of muscle tissue during the action of tensile stress (tensile force)

Legend: Hz—hertz, N/m—Newton-meter, [-]—no unit of measurement, ms—milliseconds.

**Table 2:** GM and TFL in hip OA.

Authors	TFL/GMED	Participants	Examination	Results
[1]	GMED TFL	Total N–40: HOA group N–20 patients with unilateral HOA were divided into two subgroups based on OA severity: Mild N—10 (5F/5M): -age 62.7 ± 5.9 yrs; -height: 164.2 ± 8.4 cm; -weight: 81.4 ± 18.1 kg; -BMI: 30.1 ± 5.6 kg/m^2^. Moderate-Severe N–10 (6F/4M): -age: 62.7 ± 5.9 yrs; -height: 167.6 ± 8.3 cm; -weight: 84.6 ± 18.9 kg; -BMI: 29.8 ± 5.1 kg/m^2^. Control N—20 (11F/9M): asymptomatic volunteers; -age: 62.1 ± 5.6 yrs; -height: 167.5 ± 9.6 cm; -weight: 69.7 ± 9.7 kg; -BMI: 24.8 ± 2.8 kg/m^2^.	- the Minnesota Leisure Time Physical Activity; Questionnaire - hand dynamometer; - MRI.	1. The statistical analysis showed that the GMED asymmetry observed in MRI was correlated with OA severity. 2. The Moderate-Severe clinical OA group exhibited greater GMED asymmetry than the asymptomatic group. 3. No such differences were noted in TFL.
[44]	GMED	Total N – 40 divided into two groups: Group HOA N—20 (11F/9M) patients with unilateral HOA: -age: 63.4 ± 5.4 yrs; -height: 165.8 ± 8.3 cm; -weight: 83.0 ± 18 kg; -BMI: 30.0 ± 5.2 kg/m^2^. Control N—20 (11F/9M): -age: 62.1 ± 5.6 yrs; -height: 167.5 ± 9.6 cm; -weight: 69.7 ± 9.7 kg; -BMI: 24.8 ± 2.8 kg/m^2^.	- Needle EMG; - Functional assessment.	1. The statistical analysis showed no significant differences in the variability in GMED muscle activation regardless of the tested parts (anterior, middle, and posterior). 2. During gait, the variability in the anterior hip GMED segments was significantly lower in the OA group in the early and entire stance. 3. However, the value of the average or peak GMED activity of all tested parts did not differ significantly between the groups.
[13]	GMED	Total N—40 divided into two groups: Group HOA N—20 (11F/9M) patients with unilateral HOA: -age: 63.4 ± 5.4 yrs; -height: 165.8 ± 8.3 cm; -weight: 83.0 ± 18 kg; -BMI: 30.0 ± 5.2 kg/m^2^. Control N—20 (11FK/9M): -age: 62.1 ± 5.6 yrs; -height: 167.5 ± 9.6 cm; -weight: 69.7 ± 9.7 kg; -BMI: 24.8 ± 2.8 kg/m^2^.	- Needle EMG; - Footswitches (Interlink Electronics FSRTM 402); - Step-up, step-down, and side-step tasks.	1. Step-up and step-down task test—reduced activity of the middle and posterior GMED was observed in the OA patients, compared to the control group. 2. Side-step task test—the anterior and posterior GM were characterized by later TTP.
[10]	GMED	Total N—30 Group HOA N—13 patients with unilateral HOA: -age: 51.1 ± 2.3 yrs; -height: 178.2 ± 4.3 cm; -weight: 84.2 ± 6.8 kg. Control N—17 asymptomatic volunteers: -age: 50.8 ± 1.4 yrs; -height: 173.1 ± 2.5 cm; -weight: 77.3 ± 3.8 kg.	- Force platform; - sEMG; - Step-up and step-down task; - gait assessment.	1. Higher GMED amplitude was observed during the step-up and step-down tasks in both the involved and uninvolved limbs in the OA group, compared to the control group. 2. The sEMG examination during gait exhibited significantly higher GMED amplitude in the dominant limb during the stance and swing phases in the OA group, compared to the control group. 3. The sEMG examination of the non-dominant limb during gait showed a significant increase in the amplitude only in the stance phase in the OA group, compared to the asymptomatic participants.
[45]	GMED TFL	Total N—34 Group HOA N—17 (7F/10M) patients with unilateral HOA: -age: 5.3 ± 8.3 yrs; -height: 170.2 ± 7.8 cm; -weight: 79.7 ± 14.8 kg; -BMI: 27.4 ± 3.5 kg/m^2^. Control N—17 (7F/10M) -age: 61.2 ± 7.6 yrs; -height: 172.0 ± 9.3 cm; -weight: 72.2 ± 14.5 kg; -BMI: 24.1 ± 3.3 kg/m^2^.	- VICON - motion capture system – gait analysis; - sEMG.	1. Higher activity of the TFL muscle was noted on the OA-affected side than on the unaffected side. 2. No differences in GMED activity were observed between the OA-affected and unaffected limbs. 3. No differences were found in the ASI values of GM and TFL between the OA patients and the control group. 4. TFL was shown to have a significant effect on total muscle activity. A higher %C of TFL was observed on the affected than the unaffected side. Differences in the %C of TFL were also found between the limbs of patients in the control group.
[5]	GMED TFL	Total N—40 Group HOA N—20 (11F/9M) patients with unilateral HOA: -age: 63.4 ± 5.4 yrs; -height: 165.8 ± 8.3 cm; -weight: 83.0 ± 18 kg; -BMI: 30.0 ± 5.2. Control N—20 (11F/9M) -age: 62.1 ± 5.6 yrs; -height: 167.5 ± 9.6 cm; -weight: 69.7 ± 9.7 kg; -BMI: 24.8 ± 2.8 kg/m^2^.	- hand dynamometer; - MRI.	1. No differences in the TFL volume were found between the OA-affected and unaffected sides. 2. Asymmetry was observed in the GMED volume. 3. No significant increase in fatty infiltration in GMED and TFL was found in the OA group, compared to the control group. 4. Hip abduction force was reduced in the OA group.
[46]	GMED	Total N—108 females with advanced unilateral HOA: -age: 65.4 ± 10.5 yrs; -BMI: 23.9 ± 3.6 kg/m^2^.	- hand dynamometer; - VAS; - CT.	1. The regression analysis showed that CSA and SMD of GMED were significantly correlated with the hip abduction torque in the healthy limb. 2. This correlation was not observed in the OA-affected limb.
[47]	TFL GMED	Total N—28 (7M/21F): -age: 62.32 ± 7.41 yrs; -height: 158.02 ± 6.45 cm; -weight: 55.03 ± 10.09 kg.	- MRI; - Goniometer; - hand dynamometer.	1. TFL exhibited more advanced fatty degeneration than GMAX. 2. A correlation was found between the volume and CSA of TFL. 3. The TFL strength was not correlated with CSA.
[48]	TFL GMED	- Total N—42 Group HOA (N—19) was divided into unilateral and bilateral HOA groups: Unilateral HOA group N—12 (3M/9F): -age: 62.9 ± 10.0 yrs; -height: 1.65 ± 0.09 m; -weight: 77.3 ± 14.0 kg; -BMI: 28.2 ± 3.5 kg/m^2^. Bilateral HOA group N—7 (3M/4F): -age: 63.0 ± 6.4 yrs; -height: 1.69 ± 0.14 m; -weight: 77.2 ± 15.0 kg; -BMI: 27.1 ± 3.5 kg/m^2^. Control N—23 (8M/11F): -age: 58.2 ± 8.6 yrs; -height: 1.69 ± 0.08 m; -weight: 69.9 ± 10.0 kg; -BMI: 24.4 ± 3.0 kg/m^2^.	- Isokinetic dynamometer; - MRI.	1. A significant decrease in the abduction force was observed in the HOA patients, compared to the controls. 2. No significant decrease in the TFL and GMED volume was observed.
[7]	GMED	Total N—27 (9M/18F): -age: 63.2 ± 7.6 yrs; -BMI: 28.0 ± 4.1 kg/m^2^.	- MRI; - HOOS;	CSA was not correlated with HOOS (quality of life and hip function) scores.
[49]	GMED	Total N—50 (12M/38F) with unilateral HOA: -age: 62 yrs; -BMI: 23.3 ± 4.1 kg/m^2^.	- CT; - hand dynamometer.	1. A relationship between CSA and 3D muscle volume, hip abductor muscle strength, and CT measurements was found. 2. A significant effect of GMED on hip abduction was shown.
[12]	GMED	Total N—60 HOA group divided into subgroups based on OA severity: Moderate OA: -age: 59 ± 8 yrs; -BMI: 28.7 ± 4.3 kg/m^2^. Severe OA: -age: 63 ± 8 yrs; -BMI: 30.0 ± 4.4 kg/m^2^. Control: -age: 62 ± 6 yrs; -BMI: 25.6 ± 5.0 kg/m^2^.	- sEMG; - gait analysis.	1. A reduced range of motion and increased GMED activity were demonstrated in severe HOA. 2. The hip joint function during gait deteriorated with the severity of OA.

Explanations: N—number, M—male, F—female, OA—osteoarthritis, HOA—hip osteoarthritis, yrs—years, kg—kilograms, cm—centimeters, GMED—gluteus medius, TFL—tensor fasciae latae, OA—osteoarthritis, TTP—time to peak, CSA—cross-sectional area of the muscle, SMD—skeletal muscle density, MRI—magnetic resonance imaging, CT—computed tomography, EMG—electromyography sEMG—surface electromyography, VAS—visual analog scale, ASI—asymmetry index, %C—muscle contribution to total muscle activity.

**Table 3:** Examination of GMED and TFL in patients who underwent THA.

Authors	GMED/ TFL	Participants	Examination	Results
[50]	GMED	Total N—42 (9F/33M); -age: 70.9 yrs; -BMI: 22.8 kg/m^2^.	- retrospective study; - THA was performed through a lateral approach; - CT assessment of the GM composition before the procedure; - gait speed before and 6 months after the procedure; - TUG before and 6 months after the procedure; - dynamometer before and 6 months after the procedure.	1. Changes in the GMED composition were shown to affect postoperative outcomes in patients. 2. The LDL of the gluteal muscle was shown to exert a significant effect on TUG results after 6 months.
[51]	GMED	Total N—10 (8F/2M); -age: 61.9 ± 9.4 yrs.	- posterior approach; - MRI assessment of changes in muscle length; - dynamometry during gait.	1. The length of abductor muscles, including GMED, in the THA-treated limb was shortened significantly, compared to the non-operated side. 2. The data suggest that THA may have an adverse effect on the stability of the joint.
[52]	GMED TFL	Total N—22: Group THA N—11: -age: 64.55 ± 9.53 yrs; -height: 1.67 ± 0.08 m; -weight: 74.1 ± 4.97 kg; -BMI 26.46 ± 4.49 kg/m^2^; Healthy control N—11: -age: 62.36 ± 8.15 yrs; -height: 1.68 ± 0.08 m; -BMI: 23.76 ± 1.73 kg/m^2^.	- anterior approach; - estimation of the sufficiency of a single sEMG test for assessment of the MVC of hip abductors; - sEMG; - AMTI AccuGait force plate; - examination in 2 sessions at a 1-week interval; - balance test; - examination at 45–60 days after the procedure.	1. No differences in GMED and TFL were found between the sEMG sessions. 2. Higher GMED activity was observed in the Unipodal and Bipodal balance test on both the operated and non-operated sides, compared to the healthy control. 3. Simultaneous disturbances in postural parameters were observed.
[53]	GMED	Total N—88 (49M/39F); -age: 47.3 ± 1.574 yrs; Division into three groups based on Trendelenburg test results: Normal N—23 (11M/12F) Mild N—61 (36M/25F) Severe N—4 (2M/2F)	- Hardinge access; - Trendelenburg test; - MRI; - examination before and 6 months after the procedure.	1. The GMED diameter was reduced after THA at 6-month follow-up. 2. Improved function after THA, i.e., reduction of Trendelenburg gait, was observed.
[54]	GMED TFL	Total N—73 divided into groups based on PLLD: Group with PLLD N—22 (21F/1M) -age: 66.6 ± 3.61 yrs; -height: 1.53 ± 0.05 m; -weight: 53.2 ± 8.4 kg Group without PLLD N—51 (43F/8M) -age: 67.3 ± 3.92 yrs; -height: 1.56 ± 0.06 m; -weight: 53.6 ± 9.2 kg.	- posterolateral approach; - PLLD measurement; - SWE; - VAS; - dynamometer; - assessment of pelvic position; - ROM measured using a goniometer.	1. The elasticity of the abductor muscle was significantly higher prior to THA in patients from the PLLD group. 2. The multiple regression analysis showed that the preoperative elastic modulus of abductor muscles had an impact on PLLD.
[55]	GMED	Total N—74 Division into two groups based on the occurrence of limping in patients at 6 months post THA Non-limping group N—37 -age: 56.7 ± 9.3 yrs; -BMI: 23.5 ± 4.3 kg/m^2^. Limping group N—37: -age: 64.5 ± 9.6 yrs; -BMI: 21.7 ± 2.7 kg/m^2^.	- anterolateral approach; - dynamometer; - TUG test; - CT–CSA of gluteus medius.	1. The statistical analysis showed a reduction in the CSA of GMED in patients from the limping group before THA. 2. The results suggest that the preoperative CSA of the GMED muscle predicts limping at 6 months post THA.
[56]	GMED TFL	Total N—76 THA group N—52 (28F/27M); -age: 58 ± 9.0 yrs; -weight: 77.4 ± 14.2 kg; -height: 169 ± 9 cm. Healthy control N—24 (8F/16M); -age: 54.0 ± 6.6 yrs; -weight: 75.5 ± 11.3 kg; -height: 172 ± 7 cm.	- standard lateral transgluteal (Bauer) approach; - three-dimensional ultrasonic motion analysis system (CMS S50, zebris Medical GmbH, Isny, Germany); - sEMG; - before and at 6 months post THA.	1. The motion range, bioelectrical activity, and duration and distance of gait at 6 months post THA were comparable with those of the healthy control. 2. The TFL activity in EMG was higher during loading in the THA-treated group. 3. The GMED activity in EMG was higher in the initial phase of gait in the THA-treated group. 4. Higher EMG results were observed during the first 40% and last 10% of the gait cycle.
[57]	GMED	Total N—58 (45F/13M); -age: 70.9 ± 9.5 yrs; -BMI: 23.0 ± 3.4 kg/m^2^.	- lateral approach; - CT prior to THA; - dynamometer before and at 6 months post THA; - gait speed before and at 6 months post THA	1. In the entire study group, the LMM/TM value in GMED was negatively correlated with the postoperative gait speed. 2. The LDL/TM value in GMED showed a positive relationship with postoperative improvement in gait speed. 3. The LDL value was positively correlated with postoperative improvement in gait speed. 4. In the female group, the values of LMM and LMM/TM in GMED were positively correlated with postoperative improvement in gait speed.
[58]	GMED TFL	Total N—40 (23M/17F); - age 56.35 ± 12.9 yrs.	- lateral approach; - needle EMG; - MUAP analysis; - preoperative examination and at 6- and 12 weeks post THA	1. At 6 weeks post THA, the EMG examination showed denervation of abductor muscles in 15 patients. 2. After 12 weeks, spontaneous healing was observed in 8 patients from this group. 3. The MUAP analysis showed persistent denervation in the other seven patients 4. The duration of MUAP increased in the GMED and TFL muscles after 6 weeks. 5. Significant reduction in MUAP amplitude was observed only in the GMED muscle. 6. After 12 weeks, the values were slightly different from the results obtained before THA, which indicated restoration of normal muscle activity
[62]	GMED	Total N—40 (6M/34F); - age 58 yrs.	- posterolateral approach; - CT assessment of GM volume changes at 3 weeks and 2 years post THA	The regression analysis showed that the increase in the cross-sectional area of GMED assessed by CT at 2 years post THA was associated with improved functional test results.
[60]	GMED	Total N—124F Division into two groups based on TUG results Fast Group N—103: -age: 65.3 ± 10.5 yrs; -BMI: 24.0 ± 5.1 kg/m^2^. Slow Group N—21: -age 74.4 ± 7.8 yrs; -BMI 25.3 ± 5.6 kg/m^2^.	Posterolateral modified Watson-Jones approach and direct anterior approach; - hand dynamometer before and at 1 year post THA; - CT before and at 2 weeks post THA; - TUG before and at 1 year post	1. Significantly lower abductor muscle strength was observed on the non-operated side in the slow group 2. One year after THA, the abductor muscle strength was significantly lower on the operated and non-operated side in the slow group. 3. Before THA, the CT of GMED showed significantly lower density on the non-operated and operated side in the slow group. 4. The strength and quality of GMED muscle on the healthy side, but not on the THA-treated side, are important for the outcomes of the TUG test performed at 1 year post THA.
[61]	GMED	Total N—22 (8M/14F) -age: 60 ± 14.4 yrs. Mean THA follow-up period: 13.8 ± 2.3 months	- posterolateral approach; - MRI; - HHS	1. Significant GMED fatty degeneration was observed on the operated versus the non-operated side. 2. The age correlated positively with fatty atrophy. 3. Steatosis exerts a negative effect on HHS results.
[59]	GMED TFL	Total N—10 (2F/8M); -age: 57.3 yrs.	- CT; - hip joint contact forces; - ADL—lateral approach	Compared to preoperative results, the findings at 3 months post THA were as follows: 1. An insignificant increase in total TFL volume 2. Insignificant changes in the decrease in the lean muscle volume of GMED and an increase in the lean muscle volume of TFL 3. Fatty degeneration—an insignificant increase in the GMED 4. Fatty degeneration—a significant decrease in the TFL muscle 5. The correlation suggests that fatty degeneration in TFL and GMED has a significant effect on hip joint contact forces. 6. GMED steatosis strongly correlates with hip joint contact forces while sitting down and getting up from a chair.

Explanations: N—number, M—male, F—female, yrs—years, kg—kilograms, cm—centimeters, GMED—gluteus medius, TFL—tensor fasciae latae, OA—osteoarthritis, TTP—time to peak, CSA—cross-sectional area of the muscle, SMD—skeletal muscle density, MRI—magnetic resonance imaging, CT—computed tomography, ADL—activities of daily living, HHS—Harris Hip Score, TUG—Timed Up and Go test, MUAP—motor unit action potentials, PLLD—perceived leg length discrepancy, EMG—electromyography sEMG—surface electromyography, VAS—visual analog scale, SWE—shear wave elastography, LMM/TM—lean muscle mass area/total muscle area, LDL/TM—low-density lean tissue/total muscle area, LDL—low-density lean tissue, VAS—Visual Analogue Scale, MVC—maximum voluntary contraction.

**Table 4:** Use of MyotonPRO to assess hip abductor muscles, with particular focus on GMED and TFL.

Authors	Aim of Study	Study Design	Muscles	Contraction/Rest	Conclusion
[63]	1. Assessment of differences in the effectiveness of standard LSE used in the treatment of LBP, compared with an exercise program based on a combination of LSE with muscle strengthening and stretching exercises—CE 2. Assessment of the effectiveness of new exercise programs that may be used in the treatment of LBP	The study included elderly patients suffering from LBP for at least 3 months; Total N—20 (6M/14F) (mean age 67.5 ± 5.8 yrs); the study lasted 8 weeks; The participants were divided into two 10-person groups based on the type of exercises performed 3 times a week: LSE—patients performing lumbar spine stabilization exercises: -average age (yrs)—67.3 ± 5.92; -average BMI (kg/m^2^)—23.31; CE—patients performing combined exercises: -average age (yrs)—67.7 ± 5.37; -average BMI (kg/m^2^)—22.37.	GMED	rest	1. Both groups of patients (CE and LSE) exhibited a decrease in muscle stiffness, including GMED, after the exercise period. 2. The study showed that both programs were effective in the treatment of LBP in elderly patients. 3. The comparison of the CE and LSE programs showed that the inclusion of additional programs of stretching and strengthening of the lower limb was a more effective method for LBP treatment in elderly patients.
[64]	Assessment of the relationship between lower limb muscle stiffness and CPP symptoms in females.	Patients suffering from CPP for at least 3 months were qualified for the study. The study included an asymptomatic control group. Total N—197F (age range 18–50 years). The study group was divided into two CPP and an asymptomatic group: CPP group—N—149 -average age (yrs)—35.7 ± 7.6; -average BMI (kg/m^2^)—25.71 ± 3.63. Asymptomatic—N—48 average age (yrs)—34.9 ± 9.2; average BMI (kg/m^2^)—25.1 ± 3.7.	TFL GMED	rest	1. A slight correlation was found between muscle stiffness measurements and clinical CPP indices. 2. Significantly increased stiffness was found in 5 of the 11 muscles examined in the CPP group. In the case of TFL and GMED, no statistically significant differences were observed between the groups.
[65]	Assessment of the potential effect of 6-week specialist PT on changes in muscle stiffness and the ability to perform CMJ.	Professional volleyball players were qualified for the study, which lasted 6 weeks and included a 4-week preparatory period and a 2-week period before the tournament. Total N—16M - experience—4–5 years of consistent training: -average age (yrs)—21.12 ± 1.67; -average height (cm)—191.60 ± 5.74; -average weight (kg)—86.30 ± 6.66 kg. The examination was conducted two days before the start of training and at the end of each study week.	TFL	rest	1. The study showed that the 6-week PT program did not induce a statistically significant increase in lower limb muscle stiffness, except for the tibialis anterior muscle. 2. The statistically insignificant increase in muscle stiffness was sufficient for a significant improvement in CMJ.
[66]	Assessment of the effectiveness of cryosauna in preventing DOMS. Biochemical tests of participants’ blood were performed, and markers of muscle tissue damage were determined. The stiffness of selected lower limb muscles was also examined using the MyotonPRO device.	The study included healthy individuals training in martial arts. The participants were asked to give up training 48 hours prior to the study. Total N—31 was divided into two groups: Experimental group—CRYO N—16—cryosauna was used after a series of exercises in this group: -average age (yrs)—22.1 ± 1.8; -average height (cm)—176.3 ± 8.3 cm; -average weight (kg)—76.1 ± 17.1 kg. Control group—CON N—15—participants performed the same series of exercises without cryosauna: -average age (yrs)—21.8 ± 1.6; -average height (cm)—175.3 ± 11.5 m; -average weight (kg)—76.2 ± 17.2. The participants were examined (blood sampling and MyotonPRO examination) before, immediately after, and 24, 48, 72, and 96 h after the exercise.	TFL	rest	1. The study showed that cryosauna caused a decrease in the concentration of blood biomarkers of muscle tissue damage (creatine kinase) and muscle stiffness following DOMS. 2. The stiffness degree did not change in any muscles. No differences in the stiffness of TFL were observed in the analyzed periods.
[67]	Assess the impact of outcomes of marathon running and long-term endurance training on lower limb muscle stiffness in middle-aged marathon runners.	Long-distance runners aged 50–73 years were qualified for the study. The participants reported no illnesses and were not taking any medications regularly. All participants had been training actively for at least a year. Total N—31 All male group: -average age (yrs)—57.32 ± 6.25; -average height (cm)—175.61 ± 5.74; average weight (kg)—75.36 ± 7.89 k. Stiffness was measured using a MyotonPRO device. The examination was performed three times: twice before the start: 1 day and 1–2 h, and immediately after the marathon (up to 30 min after the run). Both limbs were measured.	TFL	rest	The study did not confirm the hypothesis that muscle biomechanical properties and resting tension may change after long-term exercise.

Explanations: N—number, M—male, F—female, yrs—years, kg—kilograms, cm—centimeters, GMED—gluteus medius, TFL—tensor fasciae latae, LSE—lumbar stabilization exercise, LBP—low back pain, CE—combined exercise, CPP—chronic pelvic pain, PT—plyometric training, CMJ—countermovement jump, DOMS—delayed onset muscle soreness.

**Table 5:** Use of MyotonPRO to assess lower limb muscles in patients with OA.

Authors	Aim of Study	Study Design	Muscles	Contraction/Rest	Conclusion
[16]	1. Assessment of the properties of quadriceps muscles in dominant and non-dominant limbs in elderly female KOA patients 2. Determination of the correlation between quadriceps muscle characteristics and abnormal foot posture	Only females more than 50 years old with unilateral or bilateral KOA were qualified for the study. Total N—40 F: -average age (yrs)—65.28 ± 5.93; -average height (cm)—163.03 ± 6.37; -average weight (kg)—59.25 ± 7.31; -average BMI (kg/m^2^)—22.27 ± 2.19; unilateral KOA—N—25; bilateral KOA—N –15. The MyotonPRO examination was performed in the supine position.	RF VL VM	contraction	1. Significantly greater tension and stiffness of the quadriceps muscle were observed in the non-dominant leg, compared to the dominant limb. 2. A relationship was found between changes in quadriceps muscle efficiency and static foot posture in the elderly female patients with KOA.
[17]	1. Assessment of differences in the biomechanical parameters of quadriceps muscles in patients with knee osteoarthritis (KOA) in ultrasound examination, SWE, and MyotonPRO, compared to healthy controls 2. Identification of relationships between the examined muscle tissue and KOA severity	The study included over 60-year-old patients with unilateral KOA and healthy volunteers. Total N - 80, including: KG N—40 (13M/27F)—unilateral knee osteoarthritis; -average age (yrs)—66.78 ± 3.56; -average height (cm)—159.63 ± 6.17; -average weight (kg)—61.25 ± 4.34 -average BMI (kg/m^2^)—24.13 ± 2.39. CG N—40 (15F/25M) - control group of healthy volunteers: -average age (yrs)—66.38 ± 3.19; -average height (cm)—160.15 ± 6.08; -average weight (kg)—60.90 ± 4.62 -average BMI (kg/m^2^)—23.85 ± 2.57. The MyotonPRO examination was conducted in a neutral, relaxed supine position.	RF VM VL	rest	1. The MyotonPro examination showed asymmetry of quadriceps biomechanical parameters between the sides with and without OA in patients with unilateral KOA. 2. Quadriceps muscle stiffness was higher in the KOA patients than in the healthy controls. 3. It was observed in the study group that tension was dependent on the intensity of pain sensation (VAS) and the disability degree (WOMAC).
[68]	Assessment of the relationship between muscle tissue properties and mechanical pain sensitivity in adults with KOA	Total N—42; -average age (yrs)—67.5 ± 8.5.	RF BF	rest	The study showed that changes in the biomechanical properties of BF (weakening) may impair joint loading, which causes greater pain during movement.
[15]	Assessment of the relationship between biomechanical properties of GM and foot posture asymmetry in patients with unilateral and bilateral KOA	Over 45-year-old patients with diagnosed KOA were qualified for the study. Total N—62, including: UG (unilateral group)—N—30 (13F/17M) -average age (yrs)—62.97 ± 6.96; -average height (cm)—156.13 ± 27.20; -average weight (kg)—63.30 ± 8.42; BG (bilateral group)—N—32 (15F/17M) -average age (yrs)—60.09 ± 6.12; -average height (cm)—161.84 ± 6.27; -average weight (kg)—64.44 ± 7.40. The MyotonPRO examination was conducted in the relaxed supine position.	GL GM	rest	1. Higher stiffness and tension were observed in GL in RSL than in RML in the KOA patients. 2. Greater asymmetry in foot posture was found in the patients with unilateral KOA, compared to those with bilateral KOA. 3. The foot posture was significantly correlated with the biomechanical properties of GM and KOA severity.
[14]	1. Comparison of RF, VL, and VM stiffness between KOA patients and healthy individuals 2. Determination of potential differences in stiffness between the study groups, depending on the range of knee joint motion 3. Identification of a relationship between the stiffness of the studied muscles and impaired knee joint function in KOA patients	The study involved KOA patients and healthy controls. Both study groups were age-matched. Total N—50, including: KOA group—N—25 (18/7): -average age (yrs) 62.2 ± 8.3; -average BMI (kg/m^2^)—24.22 ± 1.96; Healthy group – N—25 (18/7): -average age (yrs)—59.44 ± 5.33; -average BMI (kg/m^2^)—23.67 ± 2.67. The measurements were taken in a sitting position with 60° and 90° flexion. The angle was controlled by an orthosis.	RF VL VM	contraction	1. Greater VL stiffness was observed in the KOA-affected patients than in the healthy controls. No significant differences in the stiffness of the other muscles were found between the groups. 2. The statistical analysis showed that the stiffness of the muscles increased with the motion range in both groups. 3. A correlation was found between the stiffness of the muscles and the disability degree in the KOA patients. This correlation was observed in particular between the VL stiffness and the disability degree determined with the WOMAC score.
[69]	Assessment of the effect of a reduced knee flexion angle on patella mobility in OA patients.	Patients with knee OA were qualified for the study. The study included two control groups: healthy elderly and healthy young individuals. Total N—50, including: KOA (OA-affected) patients—N—23: -average age (yrs)—71.7 ± 8.3; -average height (cm)—152 ± 7.4; -average weight (kg)—57.2 ± 9; -average BMI (kg/m^2^)—24.8 ± 3.7. Healthy elderly individuals—N—17: -average age (yrs) 69.7 ± 4.0; -average height (cm)—149.7 ± 4.4; -average weight (kg)—48.7 ± 6.3; -average BMI (kg/m^2^)—21.7 ± 2.6. Healthy young individuals—N—10: -average age (yrs)—21.7 ± 0.7; -average height (cm)—161.1 ± 5.1; -average weight (kg)—57.5 ± 8; -average BMI (kg/m^2^)—22.3 ± 3.9. The examination was performed in the supine position with 45° knee joint flexion. Stiffness, flexibility, and tension were measured using MyotonPRO. Patellar mobility was assessed with the use of PFA.	RF	contraction	1. Patella mobility was found to change in the OA patients. 2. There was a relationship between reduced patella mobility and reduced knee motion range. 3. Reduced flexibility was shown to affect knee mobility in OA.

Explanations: N—number, M—male, F—female, yrs—years, kg—kilograms, cm—centimeters. KOA—knee osteoarthritis, RF—rectus femoris, VL—vastus lateralis, VM—vastus medialis, BF—bicep femoris, GL—gastrocnemius lateralis, GM—gastrocnemius medialis, RSL—relatively serious leg, RML—relatively moderate leg, VAS—Visual Analogue Scale, WOMAC—Western Ontario and McMaster Universities Arthritis Index.

## Supplementary Materials

The following supporting information can be downloaded at: https://www.mdpi.com/article/10.3390/jcm14175982/s1, PRISMA 2020 Checklist. Ref. [102] is cited in Supplementary Materials.

## Abbreviations

The following abbreviations are used in this manuscript:

%C	muscle contribution to total muscle activity
ADL	activities of daily living
ASI	asymmetry index
CE	combined exercise
cm	centimeters
CMJ	countermovement jump
CPP	chronic pelvic pain
CSA	cross-sectional area of the muscle
CT	computer tomography
DOMS	delayed onset muscle soreness
EMG	Electromyography
F	female
GMAX	gluteus maximus
GMED	gluteus medius
GMIN	gluteus minimus
HHS	Harris Hip Score
kg	kilograms
LBP	low back pain
LDL	low-density lean tissue
LDL/TM	low-density lean tissue/total muscle area
LMM/TM	lean muscle mass area/total muscle area
M	male
MRI	magnetic resonance imaging
MUAP	motor unit action potentials
MVC	maximum voluntary contraction
N	number
OA	osteoarthritis
PLLD	perceived leg length discrepancy
PT	plyometric training
yrs	years
sEMG	surface electromyography
SMD	skeletal muscle density
SWE	shear wave elastography
TFL	tensor fasciae latae
THA	total hip arthroplasty
TTP	time to peak
TUG	Timed Up and Go test
VAS	Visual Analogue Scale
WHO	World Health Organization
KOA	knee osteoarthritis
RF	rectus femoris
VL	vastus lateralis
VM	vastus medialis
BF	bicep femoris
GL	gastrocnemius lateralis
GM	gastrocnemius medialis
RSL	relatively serious leg
RML	relatively moderate leg
